# The introduction of ‘No jab, No school’ policy and the refinement of measles immunisation strategies in high-income countries

**DOI:** 10.1186/s12916-019-1318-5

**Published:** 2019-05-17

**Authors:** Filippo Trentini, Piero Poletti, Alessia Melegaro, Stefano Merler

**Affiliations:** 1 Center for Information Technology, Bruno Kessler Foundation, via Sommarive, 18, 38123 Trento, Italy; 2 Carlo F. Dondena Centre for Research on Social Dynamics and Public Policies and Department of Social and Political Sciences, Bocconi University, Milan, Italy

**Keywords:** Measles elimination, Compulsory vaccination, School entry vaccination, High-income countries, Mathematical model

## Abstract

**Background:**

In recent years, we witnessed a resurgence of measles even in countries where, according to WHO guidelines, elimination should have already been achieved. In high-income countries, the raise of anti-vaccination movements and parental vaccine hesitancy are posing major challenges for the achievement and maintenance of high coverage during routine programmes. Italy and France approved new regulations, respectively in 2017 and 2018, aimed at raising immunisation rates among children by introducing mandatory vaccination at school entry.

**Methods:**

We simulated the evolution of measles immunity profiles in seven distinct countries for the period 2018–2050 and evaluated the effect of possible adjustments of immunisation strategies adopted in the past on the overall fraction and age distribution of susceptible individuals in different high-income demographic settings. The proposed model accounts for country-specific demographic components, current immunity gaps and immunisation activities in 2018. Vaccination strategies considered include the enhancement of coverage for routine programmes already in place and the introduction of a compulsory vaccination at primary school entry in countries where universal school enrolment is likely achieved.

**Results:**

Our model shows that, under current vaccination policies, the susceptible fraction of the population would remain below measles elimination threshold only in Singapore and South Korea. In the UK, Ireland, the USA and Australia either the increase of coverage of routine programmes above 95% or the introduction of a compulsory vaccination at school entry with coverage above 40% are needed to maintain susceptible individuals below 7.5% up to 2050. Although the implementation of mandatory vaccination at school entry would be surely beneficial in Italy, strategies targeting adults would also be required to avoid future outbreaks in this country.

**Conclusions:**

Current vaccination policies are not sufficient to achieve and maintain measles elimination in most countries. Strategies targeting unvaccinated children before they enter primary school can remarkably enhance the fulfilment of WHO targets.

**Electronic supplementary material:**

The online version of this article (10.1186/s12916-019-1318-5) contains supplementary material, which is available to authorized users.

## Background

In 2010, the World Health Assembly set three milestones with the objective of measles elimination. These include the increase of routine coverage with a first dose of measles-containing vaccine to ≥ 90%, the reduction of global annual incidence to less than 5 cases per million and a 95% decrease of global mortality from the 2000 estimates [1]. While substantial progress towards these goals has been documented, regional elimination targets have not been met yet [2].

Measles still represents one of the main causes of child mortality in low-income countries [1] but it now poses serious challenges also in regions where elimination was declared in the last decade [3, 4].

In 2017, measles cases reported to the World Health Organization (WHO) amounted to 173,330 worldwide and measles incidence rates were among the highest in Italy and Romania. Although measles cases reported to WHO may represent 2% of measles cases worldwide [1] and reporting rates are likely to be much higher in countries with better access to care [5], the European region has experienced a fourfold increase of reported cases compared to the previous year [6] and 35 deaths.

Current and future trends of measles epidemiology in different countries are certainly dependent on the background demographic conditions as well as on the effectiveness of past immunisation activities in reducing the susceptible fraction of the population [7]. In regions with currently high two-dose vaccination coverage, future sustained measles transmission may arise from individuals who are still susceptible as a consequence of low vaccine uptake experienced in the past [3, 7, 8]. In particular, the median age at infection observed between 2015 and 2016 in Mongolia [9] and in 2017 in Italy [3, 7] was around 27 years of age, therefore suggesting remarkable immunity gaps among adolescents and young adults.

However, even countries with a history of strong measles immunisation programmes, like South Korea, the UK and the USA, may be threatened in the near future by possible changes in public compliance to vaccination [10, 11], the sustained transmission in adjacent regions [12] or the low immunisation rates in closed communities [4, 13]. These complexities would contribute in making measles elimination difficult to be achieved and maintained also in high-income countries.

Following declining trends of routine coverage levels arisen from anti-vaccination movements and ‘hesitant compliers’ [10, 11], the Italian and French governments made MMR vaccination compulsory for children before they enter primary school [14, 15]. Similarly, the state of South Australia, following the example of the state of Victoria, proposed a new regulation to forbid the enrolment of unvaccinated children in kindergartens and daycare centres [16], also called the ‘No jab, No play’ policy.

A modelling framework aimed at uncovering measles immunity gaps in different socioeconomic settings [7] is here extended to investigate the potential impact of different policies to reinforce immunisation rates in high-income countries. In particular, we compare the effect of enhancing vaccination coverage of routine programmes with the impact of introducing compulsory vaccination at school entry on the residual measles susceptibility in the next 30 years.

The carried out analysis focuses on seven countries with a two-dose measles programme already in place and a high primary school enrolment rate [17], but characterised by different demographic conditions and vaccination histories: the USA, South Korea, Singapore, Australia, Italy, the UK and Ireland.

## Methods

Initial conditions on the fraction of susceptible and immune individuals in 2018 are estimated using a deterministic age structured transmission model introduced and calibrated in Trentini et al. [7]. Specifically, the model was parametrised, separately for each country, in such a way to reproduce different serological profiles reported for the seven countries considered at different time points. A detailed description of model structure and calibration can be found in [7].

In this work, we extend the model to simulate, for each country, how the susceptibility of the host population would change in the future, under current and additional vaccination programmes. Changes in measles immunity profiles caused by alternative immunisation strategies and coverage scenarios are simulated for the period 2018–2050, by neglecting the potential impact of the circulation of the infection to estimate the temporal changes in the overall fraction and age distribution of individuals at risk of measles infection after 2018. The fraction of residual susceptibility estimated in a given year *y* > 2018 therefore include individuals who may experience natural infection between 2018 and *y*, due to possible measles resurgence between 2018 and *y*.

Country-specific crude birth rates and age-specific mortality rates, as estimated by United Nations World Population Prospect (Additional file 1: 1.2 Demographic data), are explicitly taken into account. Country-specific routine vaccination activities along with recent adjustments adopted in some countries are simulated by mimicking schedule and coverage data reported by the World Health Organization and complemented with information available at the country level [7]. Different coverage scenarios are considered to assess the impact of an increase in vaccine uptake under current routine strategies. In addition, a vaccination policy targeting unvaccinated school age children is simulated on the basis of indications given by public health authorities and National Health Institutes of Italy and France [14, 15], where compulsory vaccination at school entry has recently been introduced (Additional file 1: 2 Additional results). Vaccination at school entry is also considered in combination with a catch-up campaign among individuals in mandatory school classes in 2018, as indicated in the guidelines associated with the new Italian law [15].

We therefore investigate (i) a baseline scenario where routine programmes and coverage levels remain unchanged as before the introduction of compulsory vaccination at school entry in Italy and Australia; (ii) a scenario where coverage levels associated with the baseline vaccination activities are assumed to vary between 60 and 100%; (iii) a scenario where baseline vaccination activities are complemented with vaccination at school entry, implemented on the basis of country-specific compulsory school programmes (Additional file 1: 2 Additional results), with coverage levels between 20 and 100%; and (iv) a scenario where baseline vaccination activities are complemented with vaccination at school entry and a catch-up campaign among 1–15 years old in 2018 with coverage levels of both strategies assumed to vary between 20 and 100%.

In our model, only individuals who have been previously vaccinated with a first dose are considered eligible for the second dose routine programme. This assumption is based on the fact that sub-optimal coverage is expected to characterise in the same way both first and second dose administration. For example, it is likely that parents of children who opposed the first vaccine administration will oppose to the second dose as well. This means that the major benefit of the two-dose programme is the reduction in the proportion of persons who remain susceptible because of primary vaccine failure, therefore increasing immunisation rates among vaccines [18]. In contrast, vaccination at school entry and catch-up campaigns aim at immunising children who were not vaccinated during routine programmes therefore increasing the overall vaccine uptake. In particular, in the model, individuals who had already had one dose of measles-containing vaccine were not considered eligible for school-entry vaccination. The considered coverage levels of school entry vaccination and catch-up campaign should be therefore interpreted as the proportion of vaccinated individuals among those who has never been vaccinated prior school entry or before the campaign itself.

Vaccine efficacy is assumed at 95% [19] and successfully immunised individuals are assumed to gain lifelong protection against measles infection.

The transmission potential of an infectious disease is defined by the basic reproduction number R_0_, which represents the average number of secondary infections generated by a typical index case in a fully susceptible population during the entire period of infectiousness. R_0_ can be used to estimate the proportion of immune individuals *p* required in a population to interrupt transmission as *p* = 1–1/*R*_0_. Although there is evidence that ranges for measles basic reproduction number may vary highly across different geographic regions due to local conditions [7, 20], available classical estimates on *R*_0_ range between 12 and 18 [21], therefore defining the fraction of successfully vaccinated individuals (*p*) required for elimination between 92 and 94%. In our analysis, we assume that persistent measles elimination is achieved only when the fraction of residual susceptible individuals is maintained under the 7.5% of the population in the medium-long term. Distance between the estimated percentage of immune individuals in 2050 and the immunity level required for measles elimination is used to provide a quantitative measure of adequateness of current and alternative policies and coverage levels, while accounting for realistic country-specific temporal changes in the age structure of the host population.

## Results

**Fig. 1:**
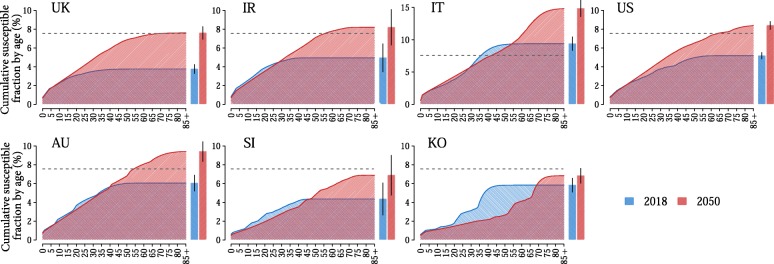
Measles susceptibility in 2018 and 2050 under baseline vaccination programmes. Cumulative fraction of susceptible individuals by age in the population in 2018 (light blue) and 2050 (light red), as estimated by assuming baseline routine country-specific vaccination activities do not change in the future. Bars refer to the total fractions of susceptible individuals in the population in 2018 (light blue) and in 2050 (light red) and vertical black lines represent their 95% credible intervals. The grey dashed line represents the 7.5% threshold required for elimination

The impact of routine vaccination activities in place before recent adjustments of immunisation policies on the amount and age distribution of residual susceptibility, as estimated for the years 2018 and 2050, is represented in Fig. 1. In the baseline scenario, coverage levels of routine programmes between 2018 and 2050 are assumed equal to those reported by WHO for 2018 [5].

The residual measles susceptibility in 2018 in the seven countries under study is estimated to lie between 3.7% (95% CI 3.2–4.2) in the UK and 9.3% (95% CI 8.3–10.4) in Italy. Remarkably, according to model estimates, in Italy, the current proportion of susceptible individuals is above the elimination threshold of 7.5%. The largest immunity gaps are found among individuals older than 10 years of age in all countries (Additional file 1: 1.3 Initial susceptibility distribution).

Coherently with findings of recent studies [7, 8], sub-optimal coverage levels experienced in the past and the progressive ageing of the population have contributed to a marked replacement of individuals who were immune as a consequence of natural infection with susceptible individuals who have been neither infected nor vaccinated. This phenomenon is expected to persist in the near future. Indeed, low measles circulation would cause the progressive replacement of the eldest cohorts of the population, who have acquired natural immunity during the pre-vaccination era, with new birth cohorts that are only partially immunised with vaccination. As a consequence, our results show that, should the coverage levels of current programmes remain unchanged, the percentage of individuals at risk of infection is expected to increase between 2018 and 2050 by more than 50% in all countries except from South Korea, where it is expected to increase by roughly 17%. This means that, by 2050, the estimated proportions of individuals at risk of infection would exceed the elimination threshold and put most of the considered countries at risk of measles outbreaks and resurgence.

Remarkably, the estimated residual susceptibility level in 2050 in Italy reaches 14.8% (95% CI 13.5–16.1) of the population, with more than 50% of susceptible individuals older than 25 years of age (see Fig. 1).

On the opposite, our results also suggest that in Singapore and South Korea, where coverage levels for routine programmes are above 95%, the estimated percentage of individuals younger than 50 years of age at risk of infection in 2050 would fall below 5%, so that measles elimination would be likely achieved and maintained in the next future.

We investigated whether baseline routine programmes are enough to achieve and maintain measles elimination. Coverage levels between 60 and 100% were considered.

**Fig. 2:**
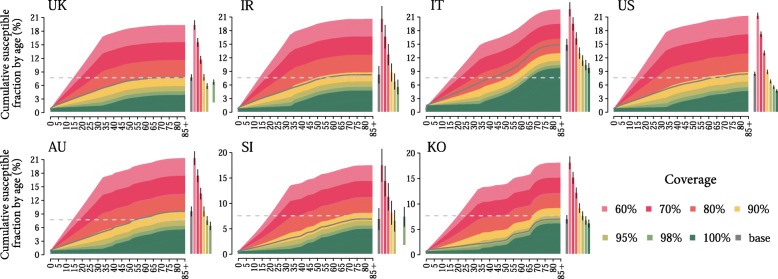
Measles susceptibility in 2050 under different routine coverage levels. Cumulative fraction of susceptible individuals by age in 2050 in the population, as estimated by assuming coverage levels of baseline routine country-specific vaccination activities between 60 and 100%. The grey line represents the estimated cumulative fraction of susceptible individuals by age in 2050, as estimated in the absence of additional vaccination programmes. Bars refer to the total fractions of susceptible individuals in the population in 2050 in different coverage scenarios, and vertical black lines represent their 95% credible intervals. The dashed grey line represents the 7.5% threshold required for elimination

We found that, in all countries with the exception of Italy, coverage levels above or equal to 95% for both first and second routine doses would allow to reach the 7.5% threshold for herd immunity (see Fig. 2). These results stress the potential of baseline routine programmes in achieving and maintaining high immunisation rates among children and are perfectly in agreement with WHO guidelines on measles immunisation. For instance, the cases of Singapore and South Korea show how small deviations from optimal immunisation rates, e.g. 90% coverage or lower, would quickly put at risk of future outbreaks even countries where high coverage levels have been already achieved in the past.

In Italy, routine vaccination programmes have the potential to dramatically mitigate the increase in measles susceptibility. However, 100% coverage in both the first and the second dose is expected to reduce the fraction of susceptible individuals in 2050 to around 10% of the overall population, and additional vaccination strategies may therefore be needed to achieve measles elimination in this country.

**Fig. 3:**
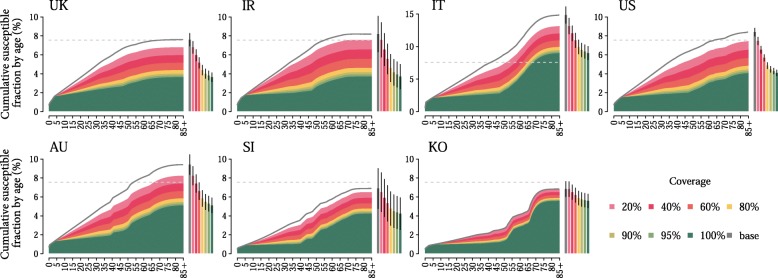
Measles susceptibility in 2050 as obtained with vaccination at school entry. Cumulative fraction of susceptible individuals by age in the population in 2050, as estimated by assuming baseline routine country-specific vaccination activities, supplemented by a new vaccination strategy at school entry and by a catch-up campaign on 1–15 years old vary. Coverage levels for the latter strategies range between 20 and 100%. The grey line represents the estimated cumulative fraction of susceptible individuals by age in 2050, as estimated in the absence of additional vaccination programmes. Bars refer to the total fractions of susceptible individuals in the population in 2050 in different coverage scenarios, and vertical black lines represent their 95% credible intervals. The dashed grey line represents the 7.5% threshold required for elimination

Temporal changes in measles susceptibility is also investigated when current routine programmes are combined with vaccination at school entry and with a catch-up campaign in 2018 on children between 1 and 15 years of age. This vaccination strategy aims at targeting unvaccinated individuals and increasing vaccine uptake and coverage levels among children. Obtained results show that for coverage levels greater than 40%, the implementation from 2018 onwards of this additional immunisation activity on top of baseline programmes can reduce the susceptible fraction of individuals below 7.5% of the population within 2050 in the UK, Ireland and the USA and Australia (Fig. 3). Interestingly, for coverage levels greater than 40%, both South Korea and Singapore would reach susceptibility levels below 3%, for individuals younger than 50 years of age, proving to be currently among the countries with the highest immunisation rates in children. The largest fraction of susceptible individuals would be found among adults, who are known to have lower contact rates and being therefore associated with a lower transmissibility potential. Similarly, in Italy, by assuming fully compliance to the recently approved vaccination law [15] (i.e. 100% coverage), vaccination at school entry would determine acceptable levels of susceptibility (around 8.9%; 95% CI 7.9–10), the largest part of which due to immunity gaps among individuals older than 50 years of age. However, our results strongly highlight that, especially for more realistic coverage levels [22], the new vaccination policy introduced in Italy may not be sufficient to prevent measles resurgence in the country.

For all countries considered, the fraction of children at risk of infection is expected to be slightly larger, with respect to results reported above, if vaccination at school entry (performed annually) was implemented without a catch-up campaign among 1–15 years old (performed only once in 2018) (Additional file 1: 2 Additional results).

## Discussion

Effective vaccination policies should be able to both mitigate the natural replenishment of susceptible individuals due to new births and reduce the residual susceptibility among individuals who escaped both routine vaccination programmes and natural infection in the past. After decades of universal vaccination of children, further enhancements of measles immunisation rates may require country-specific vaccination strategies taking into account immunity gaps expected across different socio-demographic settings and country-specific difficulties in implementing different vaccination programmes [3, 5–7, 23]. The multi-country perspective adopted in our study allows to investigate the potential impact of current and alternative vaccination programmes across countries with different immunisation histories and demographic components and to identify the feasibility of measles elimination goals [1] within 2050. Vaccination strategies considered in this study are motivated by the stagnation of vaccination coverage levels for routine programmes observed in high-income countries and laws approved in Italy and in France in 2017 and 2018 [14, 15], requiring parents to vaccinate their children against measles infection by school entry.

Our results suggest that most of countries would strongly benefit from the introduction of compulsory vaccination at school entry in addition to current routine immunisation programmes. In particular, we found that this strategy would allow the UK, Ireland and the USA to reach stable herd immunity levels in the next decades and therefore the achievement of persisting measles elimination.

Countries like Singapore and South Korea, who have been able to reach high coverage levels among children, are expected to maintain low levels of residual susceptibility both in children and young adults. South Korea in particular has reached a high level of herd immunity thanks to past immunisation policies, so that additional strategies seem not to be currently required. However, a careful surveillance is needed even in these countries, as clustering of susceptible people and small deviations of current vaccine uptake could still trigger measles resurgence [12].

In high-income countries where typically a larger fraction of susceptible individuals is found across elder ages, the strengthening of routine immunisation efforts at younger ages is fundamental but may not be enough to achieve measles elimination. This is the case of Italy, where more than 70% of cases occurred during the recent large measles outbreak has been recorded among individuals older than 15 years of age [3]. Although the importance of the compulsory school vaccination law approved last year is indisputable [24] and coverage levels for routine campaign have increased in the last months [22], the interruption of measles circulation would also require further efforts to reduce susceptibility in older age groups [3, 7].

The focus of our work is on the potential impact of immunisation strategies in reducing the proportion of individuals at risk of infection in the future. As such, in our analysis, we did not consider measles transmission between 2018 and 2050. Although the occurrence and magnitude of future measles epidemics are largely uncertain and difficult to predict [25], it is worth stressing that sufficiently high level of susceptibility in the population can promote measles circulation before 2050, therefore reducing the fraction of susceptible individuals in the host population. Our estimates of the residual susceptibility over time should be therefore carefully interpreted as representing, for each year considered, the fraction of individuals who either is still susceptible to measles infection or has experienced a natural infection after 2018.

The threshold defining measles elimination is chosen under the assumption of homogeneous mixing; therefore, it does not account for the heterogeneity of contact patterns among different age strata. In particular, for those countries where a large fraction of residual susceptibility is expected among adults, as in Italy, measles elimination may be achieved even when the proportion of susceptible individuals is larger than 7.5% of the population. In addition, our model does not take into account spatial heterogeneities in measles susceptibility as possibly resulting from different vaccination coverage at sub-national level. However, it has been recently shown that the assumption of spatially homogeneous coverage could potentially lead to underestimate the effective reproduction number [26]. Therefore, the achievement of the 7.5% susceptibility threshold may not be enough to sustain measles elimination in settings characterised by heterogeneous vaccine uptake levels.

Moreover, our estimates were obtained under the assumption that, at birth, all individuals are protected by maternal antibodies, and that there is no vaccine waning immunity. Although it is likely that children born from susceptible mothers have no maternal protection against the infection, we show that the robustness of our results is not affected by this assumption (Additional file 1: 1.5 Sensitivity analysis on maternal antibodies protection). On the other hand, although sporadic measles cases have been documented in adults who received two doses of vaccine decades before the onset of disease [3], cases among vaccinated individuals may also arise as a consequence of vaccine failure after multiple-doses administration, and less than 2% of individuals is proven to loose protective measles immunity per decade [27].

Finally, in our model, children who had already had one dose of measles-containing vaccine were not considered eligible for school-entry vaccination. Although-in principle–children who had only had one previous dose of vaccine may be also targeted by vaccination at school entry and two doses of vaccines may be administered to individuals who have never been vaccinated, the assumption we made may apply to a broader set of countries and epidemiological conditions, highlighting the potential impact of school entry vaccination under a more conservative scenario.

## Conclusions

We believe our findings contribute to the ongoing discussion on the most effective ways to achieve measles elimination goals and stress the importance of considering adjustments of current immunisation strategies, especially in countries where these appear underperforming. Recent policies aimed at increasing childhood immunisation rates through the introduction of compulsory vaccination are certainly producing positive effects, by raising the proportion of children protected against measles [24]. However, additional efforts designed specifically for each country should also be put in place to successfully achieve and maintain measles elimination in the medium-long term.

## Additional file

Additional file 1: Supporting_information.pdf. Model details, sensitivity analysis, and additional results. (PDF 5121 kb)

## Abbreviations

CI: Credible intervals
MMR: Measles, Mumps and Rubella Vaccine
R_0_: Basic reproduction number
WHO: World Health Organisation
