# Supplementary material for: The introduction of ‘No jab, No school’ policy and the refinement of measles immunisation strategies in high-income countries
Source: BMC Med. 2019 May 17;17:86. doi: 10.1186/s12916-019-1318-5 (PMC6524211; doi:10.1186/s12916-019-1318-5)
Supplement: Supplementary file 1 — Supporting_information.pdf. Model details, sensitivity analysis, and additional results. (PDF 5121 kb) [file 12916_2019_1318_MOESM1_ESM.pdf]

The introduction of "No jab, No school" policy and the refinement of measles immunisation strategies in high-income countries

Filippo Trentini[1], Piero Poletti[1], Alessia Melegaro[2] and Stefano Merler[1]

[1] Center for Information Technology, Bruno Kessler Foundation, Trento, Italy.
[2] Carlo F. Dondena Centre for Research on Social Dynamics and Public Policies and Department of Social and Political Sciences, Bocconi University, Milan, Italy

Contents

1. Materials and methods
   1.1. The non-stationary age-structured vaccination model
   1.2. Demographic data
   1.3. Initial susceptibility distribution
   1.4. Vaccination at school entry
   1.5. Sensitivity analysis on maternal antibodies protection

2. Additional results

1. Materials and methods

1.1 The non-stationary age-structured vaccination model

The evolution of susceptibility is simulated through a deterministic non-stationary age-structured model stratified in 85-years age classes. The population is divided into different compartments: individuals protected by maternal antibodies (M), susceptible individuals (S), and individuals who acquired immunity against measles either through vaccination or natural infection occurred before 2018. Among susceptible individuals we kept trace of vaccinated individuals who were not successfully immunized due to first or second dose vaccine failure of (F).
Newborn individuals are protected against measles infection for 6 months on average by the passive transfer of maternal immunity after which they become susceptible.
In the model individuals are vaccinated with a first and second dose, if any was scheduled in 2017, according with data reported on immunization schedules and coverage levels[1], by mimicking country-specific vaccination activities performed in 2017.
It is worth noting that only individuals who were not successfully immunized with a first dose (F) are considered eligible for a second dose. In contrast, vaccination at school entry and catch up campaigns are designed for children who were not reached by first or second dose vaccination programs and are therefore administered to susceptible individuals except from vaccine failures (S-F).
The model takes into account the vital dynamics of the host population, and is informed by country-specific crude birth rate and age-specific mortality rates predicted for the period considered[2].
Epidemiological transitions for each individual's age are described by the following system of ordinary differential equations:

$$
\begin{cases}
\dfrac{dM(a,t)}{dt} = \delta_{a,0}\, b(t) \displaystyle\sum_{j=0}^{85} N(j,t) - \mu M(a,t) - \delta_{a,a_1} c_1(t) M(a,t) - d(a,t) M(a,t) \\
\dfrac{dS(a,t)}{dt} = \mu M(a,t) - \left[\delta_{a,a_1} c_1(t) + \displaystyle\sum_{j=a_{CU1}}^{a_{CU2}} \delta_{a,j} c_{CU}(t) + \delta_{a,a_{SE}} c_{SE}(t) \right] S(a,t) \\
\qquad -d(a,t) S(a,t) \\
\dfrac{dR(a,t)}{dt} = \varepsilon(a) \left[\delta_{a,a_1} c_1(t) + \displaystyle\sum_{j=a_{CU1}}^{a_{CU2}} \delta_{a,j} c_{CU}(t) + \delta_{a,a_{SE}} c_{SE}(t) \right] S(a,t) + \varepsilon(a) \left[\delta_{a,a_1} c_1(t) M(a,t) + \delta_{a,a_2} c_2(t) F(a,t) \right] - \\
\qquad -d(a,t) R(a,t) \\
\dfrac{dF(a,t)}{dt} = \big(1-\varepsilon(a)\big) \left[\delta_{a,a_1} c_1(t) + \displaystyle\sum_{j=a_{CU1}}^{a_{CU2}} \delta_{a,j} c_{CU}(t) + \delta_{a,a_{SE}} c_{SE}(t) \right] S(a,t) + \big(1-\varepsilon(a)\big) \delta_{a,a_1} c_1(t) M(a,t) - \varepsilon(a) \delta_{a,a_2} c_2(t) F(a,t) - d(a,t) F(a,t) \\
N(a,t) = M(a,t) + S(a,t) + R(a,t) + F(a,t)
\end{cases}
$$

where t and a represent time and individuals' chronological age respectively; $b(t)$ and $d(a,t)$ are the crude birth rate and age-specific mortality rate at time t respectively. Coverage levels at time t associated with first dose, second dose programs and school entry vaccination are denoted by $c_1(t)$, $c_2(t)$ and $c_{SE}(t)$ respectively; a_1, a_2 and a_{SE} are respectively the age at first, second and school entry doses; $c_{CU}(t)$ is the vaccination coverage characterizing the catch up campaign performed in 2018 and targeting ages between $a_{CU1}(t)$ and $a_{CU2}(t)$; $\varepsilon(a)$ represents the vaccine efficacy, which is assumed to be 95% when the vaccine is administered at 15 months or more and 85% otherwise[3,4]. Finally, $N(a,t)$ represents the total population of age a at time t and $\delta_{a,j}$ is the Dirac delta function, which is equal to 1 for $a=j$ and 0 otherwise.

1.2 Demographic data

The model was informed with country specific longitudinal data on crude birth rates (Fig. S1), age specific mortality rates (Fig. S2 and S3), as predicted by the United Nations World Population Prospect[2] for the period 2018-2050.
In particular, according to UN predictions, in countries like Ireland, Italy, Singapore and South Korea, populations are still ageing as a result of the strong decline of fertility rates occurred

between 1960-2050. Finally, as shown in Fig.S3 in the UK, US and Australia, the population age structure is expected to remain rather stable between 2018 and 2050.

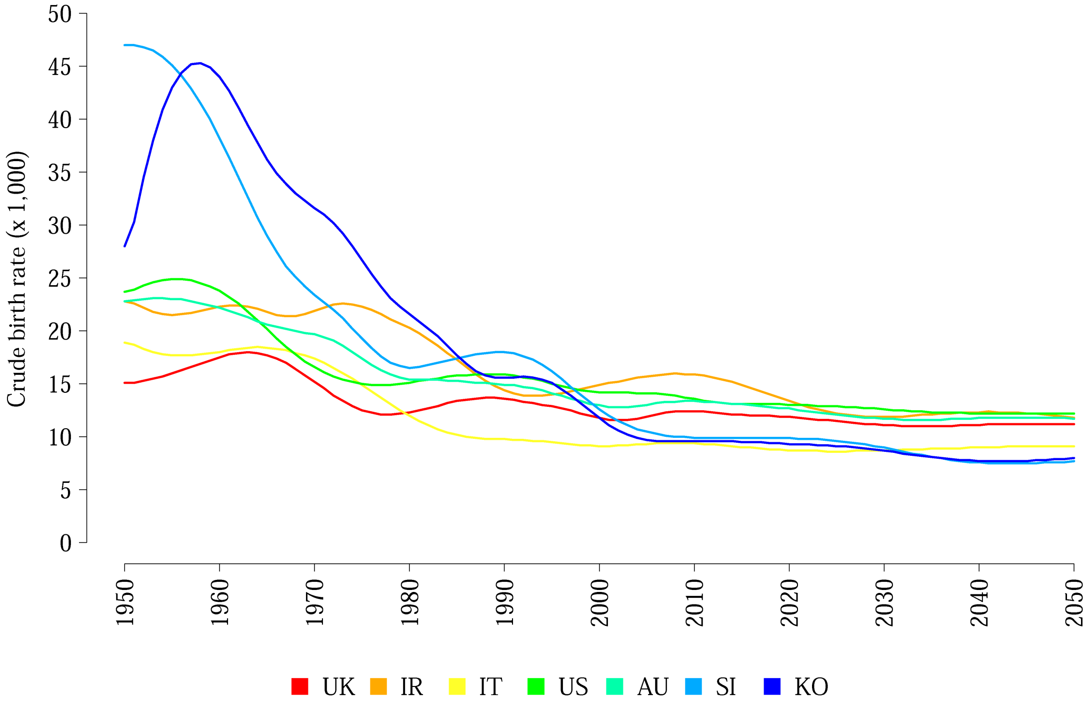

Fig. S1 Crude birth rate over time, across different countries as provided by the United Nations World Population Prospect[2].

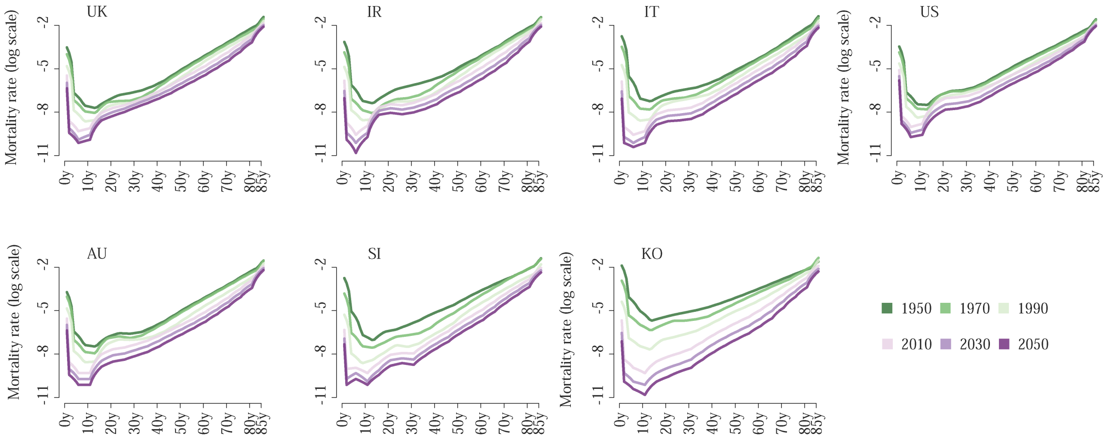

Fig. S2 Country specific mortality rates by age at different time points as provided by the United Nations World Population Prospect[2].

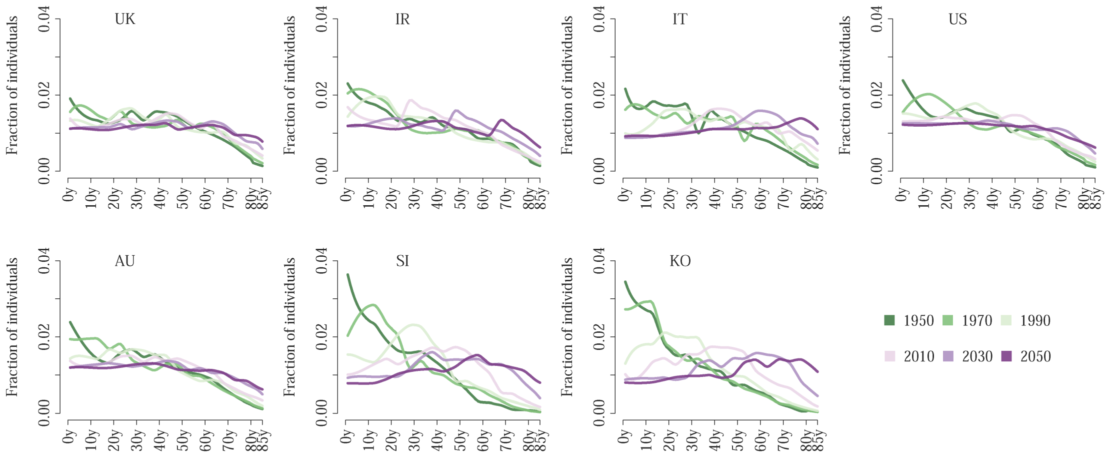

Fig. S3 Expected evolution of the population age structure over time, as obtained with the proposed demographic model by using estimates provided by the United Nations World Population Prospect[2].

1.3 Initial susceptibility distribution

In a previous modeling work[4], Trentini et al. estimated the temporal changes in the age-specific measles immunity profiles between 1950-2017 in different countries. Estimates obtained in Trentini et al.[4] for 2015 are here adopted as initial conditions on the age distribution and overall immunity level at the end of 2017, across the 7 high-income countries. The initial age distribution of susceptible individuals here considered is shown in Fig. S4.

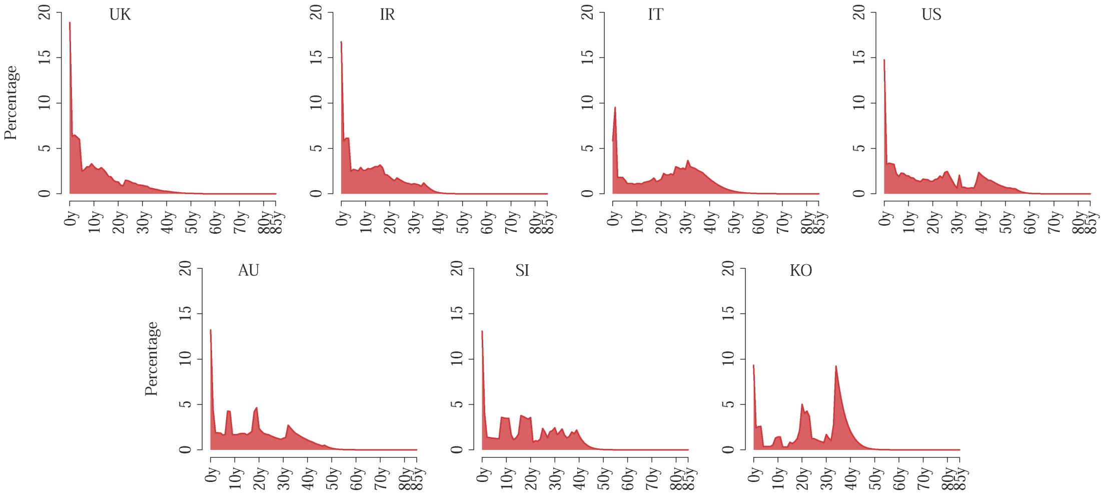

Fig. S4 Age distribution of residual measles susceptibility at the end of 2017, across different countries considered.

1.4 Vaccination at school entry

Coherently with laws approved in some European countries [5,6] on compulsory immunization, we simulated a vaccination at primary school entry aimed for all individuals who are not compliant to country-specific programs. Ages at which this new routine dose is administered are country-specific and depends on the age at primary school entry (see Tab. S1).

Table S1. Ages at primary school entry

Country	Age at primary school entry	Reference
Australia	5 years	7
Ireland	6 years	8
Italy	6 years	9
South Korea	6 years	10
Singapore	6 years	11
UK	5 years	12
US	6 years	13

1.5 Sensitivity analysis on maternal antibodies protection

In our model we assume that all children are protected by maternal antibodies at birth for an avarage period of 6 month. Due to uncertainty of seroprevalence of mothers we decided to conduct a sensitivity analysis to check for the robusteness of our results. In Fig. S5 we show projection of residual susceptibility in 2050 with the conservative assumption that all newborns end up in the susceptible class (absence of maternal protection). Estimates of residual susceptibility vary by only 4% in Australia and US, and by 1% in all other countries with respect to those obtained with our assumption on maternal antibodies. Results seems therefore to be robust to a variation in this particular assumption.

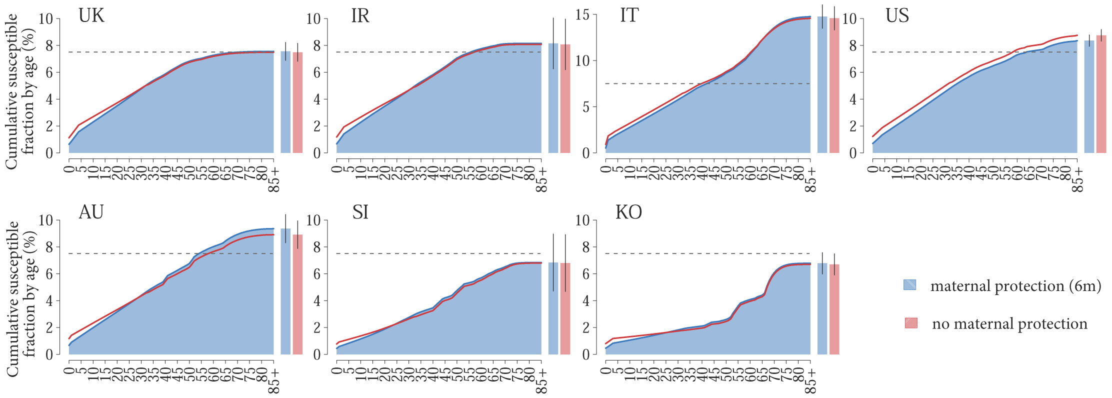

Fig.S5 Cumulative fraction of susceptible individuals by age in the population in 2050, as estimated by assuming baseline routine country-specific vaccination activities do not change in the future and i) no maternal protection (light red) ii) maternal protection for 6 months on average (light blue). Bars refer to the total fractions of susceptible individuals in the population in 2050 with the two assumptions and vertical black lines represent their 95% credible intervals. The grey dashed line represents the 7·5% threshold required for elimination.

2. Additional results

In the main text we presented results obtained by simulating current routine programs complemented with both school entry vaccination and a catch up campaign in 2018 targeting individuals between 1 and 15 years of age. In this section, temporal changes in measles susceptibility are investigated when current routine programs are combined with vaccination at school entry alone.
Obtained results show that, under coverage levels above 40%, the introduction of this additional immunization activity in 2018 can reduce the susceptible fraction of individuals below 7·5% of

the population within 2050 in UK, Ireland and US, South Korea, Singapore and Australia therefore making possible both the achievement and maintenance of measles elimination in these countries (Fig. S6). In Italy, 100% coverage vaccination at school entry would determine acceptable levels of susceptibility, but that would result still above the elimination threshold of 7·5%.

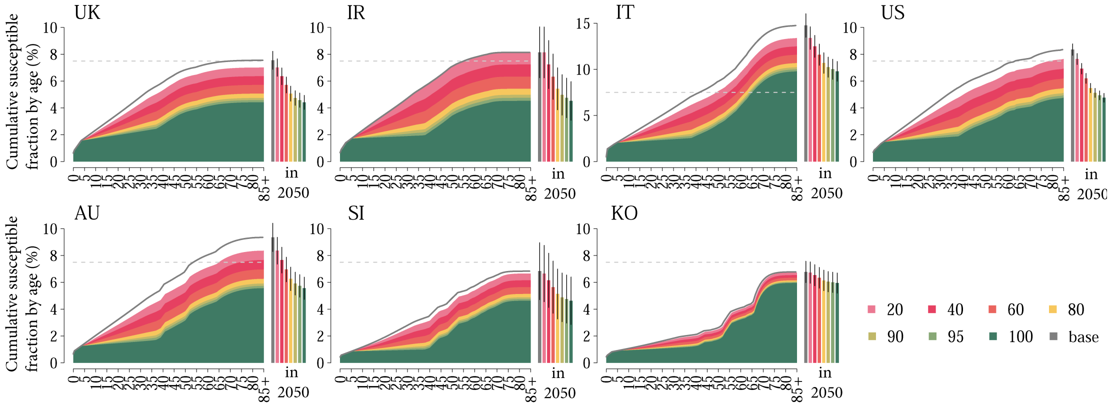

Fig.S6 Cumulative fraction of susceptible individuals by age in the population in 2050, as estimated by assuming baseline routine country-specific vaccination activities, supplemented by a new vaccination strategy at school entry. Coverage levels for the latter strategies ranges between 20% and 100%. The grey line represents the estimated cumulative fraction of susceptible individuals by age in 2050, as estimated in the absence of additional vaccination programs. Bars refer to the total fractions of susceptible individuals in the population in 2050 in different coverage scenarios and vertical black lines represent their 95% credible intervals. The dashed grey line represents the 7·5% threshold required for elimination.

1. http://www.who.int/immunization/monitoring_surveillance/data/en/ (accessed August 23, 2018)

2. http://esa.un.org/unpd/wpp/DataQuery/ (accessed August 23, 2018)
3. De Serres G, Boulianne N, Meyer F, Ward B. Measles vaccine efficacy during an outbreak in a highly vaccinated population: incremental increase in protection with age at vaccination up to 18 months. *Epidemiology and infection* 1995; **115**(02): 315–323.
4. Trentini F, Poletti P, Merler S, Melegaro A. Measles immunity gaps and the progress towards elimination: a multi-country modelling analysis. *The Lancet Infectious Diseases* 2017; **17**(10): 1089-1097.
5. Law 119/2017 - GU Serie Generale n.182 del 05-08-2017
6. Legifrance. Décret n° 2018-42 du 25 janvier 2018 relatif à la vaccination obligatoire. [Decree n° 2018-42 of 25 January 2018 regarding mandatory vaccination]. Official journal of the French Republic. 26 Jan 2018. French. https://www.legifrance.gouv.fr/eli/decret/2018/1/25/SSAP1732098D/jo/texte (accessed June 8, 2018)
7. www.education.wa.edu.au/web/our-schools/enrolling-in-school (accessed June 8, 2018)
8. www.education.ie/en/The-Education-System/Primary/ (accessed June 8, 2018)
9. www.istruzione.it/urp/obbligo_scolastico.shtml (accessed June 8, 2018)
10. transferwise.com/gb/blog/south-korean-education-overview (accessed June 8, 2018)
11. www.moe.gov.sg/education/education-system/compulsory-education (accessed June 8, 2018)
12. www.gov.uk/government/uploads/system/uploads/attachment_data/file/219167/v01-2012ukes.pdf (accessed June 8, 2018)
13. nces.ed.gov/programs/digest/d15/ch_1.asp (accessed June 8, 2018)
